# Protective Role of Interleukin-10 in Ozone-Induced Pulmonary Inflammation

**DOI:** 10.1289/ehp.1002182

**Published:** 2010-09-08

**Authors:** Gillian S. Backus, Reuben Howden, Jennifer Fostel, Alison K. Bauer, Hye-Youn Cho, Jacqui Marzec, David B. Peden, Steven R. Kleeberger

**Affiliations:** 1 National Institute of Environmental Health Sciences, Laboratory of Respiratory Biology, National Institutes of Health, Department of Health and Human Services, Research Triangle Park, North Carolina, USA;; 2 University of North Carolina–Charlotte, Department of Kinesiology, Charlotte, North Carolina, USA;; 3 Michigan State University, Department of Pathobiology and Diagnostic Investigation, Center for Integrative Toxicology, East Lansing, Michigan, USA;; 4 Center for Environmental Medicine, Asthma and Lung Biology, Department of Pediatrics, and Division of Immunology and Infectious Disease, University of North Carolina–Chapel Hill School of Medicine, Chapel Hill, North Carolina, USA

**Keywords:** air pollution, gene array, IL-10, inflammation, lung, ozone, pulmonary

## Abstract

**Background:**

The mechanisms underlying ozone (O_3_)-induced pulmonary inflammation remain unclear. Interleukin-10 (IL-10) is an anti-inflammatory cytokine that is known to inhibit inflammatory mediators.

**Objectives:**

We investigated the molecular mechanisms underlying interleuken-10 (IL-10)–mediated attenuation of O_3_-induced pulmonary inflammation in mice.

**Methods:**

*Il10*-deficient (*Il10*^−/−^) and wild-type (*Il10*^+/+^) mice were exposed to 0.3 ppm O_3_ or filtered air for 24, 48, or 72 hr. Immediately after exposure, differential cell counts and total protein (a marker of lung permeability) were assessed from bronchoalveolar lavage fluid (BALF). mRNA and protein levels of cellular mediators were determined from lung homogenates. We also used global mRNA expression analyses of lung tissue with Ingenuity Pathway Analysis to identify patterns of gene expression through which IL-10 modifies O_3_-induced inflammation.

**Results:**

Mean numbers of BALF polymorphonuclear leukocytes (PMNs) were significantly greater in *Il10*^−/−^ mice than in *Il10*^+/+^ mice after exposure to O_3_ at all time points tested. O_3_-enhanced nuclear NF-κB translocation was elevated in the lungs of *Il10*^−/−^ compared with *Il10*^+/+^ mice. Gene expression analyses revealed several IL-10–dependent and O_3_-dependent mediators, including macrophage inflammatory protein 2, cathepsin E, and serum amyloid A3.

**Conclusions:**

Results indicate that IL-10 protects against O_3_-induced pulmonary neutrophilic inflammation and cell proliferation. Moreover, gene expression analyses identified three response pathways and several genetic targets through which IL-10 may modulate the innate and adaptive immune response. These novel mechanisms of protection against the pathogenesis of O_3_-induced pulmonary inflammation may also provide potential therapeutic targets to protect susceptible individuals.

Ozone (O_3_) is the most toxic oxidant gas in air pollution mixtures and has been associated with numerous health effects, including airway inflammation and hyperreactivity (Plopper et al. 1998; Toward and Broadley 2002), exacerbation of asthma (McConnell et al. 2002; Neuhaus-Steinmetz et al. 2000), and increased mortality and morbidity (Jerrett et al. 2009). O_3_-induced lung inflammation is characterized by polymorphonuclear leukocyte (PMN) infiltration (Mudway and Kelly 2000) and release of a number of proinflammatory cytokines, such as tumor necrosis factor-α (TNF-α) (Bhalla et al. 2002; Cho et al. 2001), interleukin-6 (IL-6) (Johnston et al. 2005), and the PMN chemoattractant macrophage inflammatory protein 2 (MIP-2) (Driscoll et al. 1993). O_3_ causes upregulation of inducible nitric oxide synthase (iNOS), which further contributes to lung injury (Fakhrzadeh et al. 2002; Inoue et al. 2000; Kleeberger et al. 2001). Lung injury after O_3_ exposure may directly or indirectly affect adaptive immune responses such as T-cell proliferation (Jakab et al. 1995), response to allergen (Depuydt et al. 2002), and upregulation of costimulatory molecules such as cellular differentiation factor 86 (CD86, B7.2) that contribute to T-cell activation (Koike et al. 2001).

Interleuken (IL)-10 is a pleiotropic cytokine that is produced by activated monocytes, macrophages, and helper T-cells, and B-cells. IL-10 reduces TNF-α and IL-6 production (Lang et al. 2002), and inhibits MIP-2 (Standiford et al. 1995). Previously, Reinhart et al. (1999) showed that intratracheal instillation of recombinant IL-10 in Sprague-Dawley rats before O_3_ exposure significantly reduced O_3_-induced PMN infiltration and pulmonary hyperpermeability responses [fibronectin, albumin, bronchoalveolar lavage fluid (BALF) protein]. However, the relationship between IL-10 and other inflammatory mediators and downstream molecular events has not been investigated. IL-10 inhibits inflammation by suppressing macrophage CD86 expression leading to anergic T-cells in a schistosomiasis model (Ding et al. 1993; Flores Villanueva et al. 1994). IL-10 also inhibits iNOS production in macrophages (Cunha et al. 1992) and blocks nuclear factor-κB (NF-κB) activation (Saadane et al. 2005). Tyrosine phosphorylation of the intracellular domains of the IL-10 receptor is known to activate the signaling transducer and activator of transcription-3 (STAT3) pathway (Donnelly et al. 1999). Tarzi et al. (2006) observed that peptide immunotherapy caused induction of IL-10 and suppressor of cytokine signaling 3 (SOCS3) in a bee venom model, and Gao and Ward (2007) has implicated SOCS3 in inflammatory diseases.

Polymorphisms in the human *IL10* gene have been associated with the development of asthma (Chatterjee et al. 2005) and lipopolysaccharide (LPS) sensitivity (Schippers et al. 2005). The functional role of pulmonary IL-10 has been studied in experimental animals in response to LPS (Standiford et al. 1995), silica (Huaux et al. 1998), and infectious organisms (Higgins et al. 2003) and as a key mediator in allergy (Akdis et al. 2001) and cystic fibrosis (Saadane et al. 2005). However, the role of IL-10 in O_3_-induced lung inflammation and its underlying mechanism has not been sufficiently studied.

In this study, we tested the hypothesis that targeted deletion of *Il10* in mice would enhance O_3_-induced pulmonary inflammation via modulation of expression of inflammatory mediators CD86 and MIP-2 and nuclear transcription factors NF-κB and STAT3. We compared O_3_-induced alterations in pulmonary injury phenotypes and putative downstream molecular events between *Il10*-sufficient (*Il10*^+/+^) and *Il10*-deficient (*Il10*^−/−^) mice. We also used global mRNA expression analyses of lung tissue to identify patterns of gene expression that are modulated by IL-10 after exposure to O_3_. Enhanced O_3_-induced inflammation phenotypes in *Il10*^−/−^ mice compared with *Il10*^+/+^ mice were consistent with a protective role for IL-10. Microarray and pathway analyses identified significant differences in inflammatory mediator expression between *Il10*^+/+^ and *Il10*^−/−^ mice in response to O_3_ and suggested novel genetic targets [e.g., cathepsin E (*Ctse*) and serum amyloid A3 (*Saa3*)] affiliated with *Il10* expression and response to environmental oxidant exposure.

## Materials and Methods

### Animals

We purchased male *Il10*^+/+^ (C57BL/6) and *Il10*^−/−^ (B6.129P2-*Il10^tm1Cgn^*/J) mice (6–8 weeks) from Jackson Laboratories (Bar Harbor, ME). We provided mice with water and pelleted open-formula rodent diet NIH-31 (Zeigler Brothers, Gardners, PA) ad libitum. All experimental procedures were conducted in accordance with approved guidelines from the National Institutes of Health (Institute of Laboratory Animal Resources 1996) and the American Physiological Society (2002). Animals were treated humanely and with regard for alleviation of suffering.

### O_3_ exposure

We placed mice in individual stainless steel wire cages within a Hazelton 1000 chamber (Lab Products, Maywood, NJ) equipped with a charcoal and high-efficiency particulate air–filtered air supply. We exposed mice to 0.3 ppm O_3_ or filtered air for 24, 48, or 72 hr (23.5 hr/day) as described previously (Cho et al. 2007).

### Necropsy and BALF analyses

We euthanized mice (intraperitoneal sodium pentobarbital, 104 mg/kg) immediately after exposure. We lavaged the right lung with Hanks’ balanced salt solution and processed the lung for cell and total protein (a marker of lung permeability) analyses following Cho et al. (2001). The left lung was snap-frozen in liquid nitrogen for molecular analyses.

### Histological analysis

We inflated lavaged right lungs with 10% formalin, removed en bloc, and immersed the lungs in 10% formalin. Details of histological analyses are in the Supplemental Material (doi:10.1289/ehp.1002182). Immunohistological staining was done using a specific antibody against Ki-67 (ab15580; Abcam, Cambridge, MA); we followed the procedures outlined by Cho et al. (2007).

### Real-time quantitative reverse-transcriptase polymerase chain reaction

We isolated total RNA from left lung homogenates using the RNeasy Midi Kit (Qiagen Inc., Valencia, CA) following the manufacturer’s instructions and as described by Cho et al. (2007). Details of quantitative reverse-transcriptase polymerase chain reaction (RT-PCR) procedures are outlined in the Supplemental Material (doi:10.1289/ehp.1002182).

### Western blot analysis

We prepared total lung protein in RIPA buffer containing protease and phosphatase inhibitors. Details of Western blot procedures and analyses are presented in the Supplemental Material (doi:10.1289/ehp.1002182).

### Enzyme-linked immunosorbant assay for MIP-2 and NF-κB subunit, p65

We used 50 μL BALF from each mouse for enzyme-linked immunosorbant assay (ELISA). The procedures were performed following manufacturer’s instructions (R&D Systems, Minneapolis, MN). Processing and analysis procedures are detailed in the Supplemental Material (doi:10.1289/ehp.1002182).

### Nuclear protein isolation and electrophoretic mobility shift assay for NF-κB

We prepared nuclear extracts from right lung lobes using a Nuclear Extraction Kit (Active Motif, Carlsbad, CA). Details of the electrophoretic mobility shift assay (EMSA) procedures are in the Supplemental Material (doi:10.1289/ehp.1002182).

### Gene array analysis

#### RNA isolation and Affymetrix GeneChip hybridization

We isolated total RNA from left lung lobes, and the RNA was used after passing quality testing using an Agilent Bioanalyzer 2100 (Agilent Technologies, Inc., Santa Clara, CA, USA). For each treatment group, we performed GeneChip analysis (Affymetrix, Santa Clara, CA) analyses in duplicate (air controls) or triplicate (O_3_ exposed). Further details of RNA processing and hybridization are in the Supplemental Material (doi:10.1289/ehp.1002182).

#### Data analysis

We normalized and summarized the resulting files (in CEL format) with the Robust Multichip Average method using RMAExpress (http://rmaexpress.bmbolstad.com/). We exported log2 values for analysis using the Spotfire DecisionSite (TIBCO Spotfire, Somerville, MA). Hierarchical clustering identified one outlier, a sample from an *Il10*^−/−^ mouse exposed to O_3_ for 24 hr, which was not included in subsequent analysis. We also applied Ingenuity Pathway Analysis (IPA) software (Ingenuity Systems, Inc., Redwood City, CA), a structured network knowledge-based approach, to evaluate functions and to elect putative interaction and signaling mechanisms through which IL-10 plays a role in pulmonary pathogenesis in response to O_3_. We further analyzed statistically the CEL files by two-way analysis of variance (ANOVA; *p* < 0.01) using genotype (*Il10*^+/+^, *Il10*^−/−^) and exposure (air, 24-hr O_3_, 48-hr O_3_, 72-hr O_3_) as the variables in GeneSpring GX 11.0 Expression Analysis software program (Agilent Technologies). We deposited the raw data discussed in this publication into the National Center for Biotechnology Information’s Gene Expression Omnibus data repository (http://www.ncbi.nlm.nih.gov/geo/, series GSE25095) and the National Institute of Environmental Health Sciences Chemical Effects in Biological Systems database (http://cebs.niehs.nih.gov/, accession no. 005-00003-0070-000-5). Details of the processes used to identify genes with altered transcript levels as a function of exposure, strain, and/or time are in the Supplemental Material (doi:10.1289/ehp.1002182). After consolidating probe sets, we loaded the resulting list of 165 genes and annotation into IPA (version 7.4, April 2009) to identify unique gene expression pathways that reflected an interaction between strain and treatment effect.

### Statistics

We expressed all data as group means ± SE. We log-transformed PMN data to ensure normal data distribution and equal variance. We assessed differences in effects of O_3_ and IL-10 on response phenotypes by two-factor ANOVA. The factors were exposure (air or O_3_) and genotype (*Il10*^+/+^ or *Il10*^−/−^). Dependent variables were BALF protein concentration, BALF cells, mRNA expression, and protein levels. We used the Student Newman–Keuls post hoc test to compare group means. We performed all statistical analyses using the SigmaStat statistics package (version 3; Systat Software, Inc., Point Richmond, CA). We accepted statistical significance at *p* < 0.05. Sample sizes are included in the figure captions.

## Results

### Lung inflammatory responses

**Figure 1:**
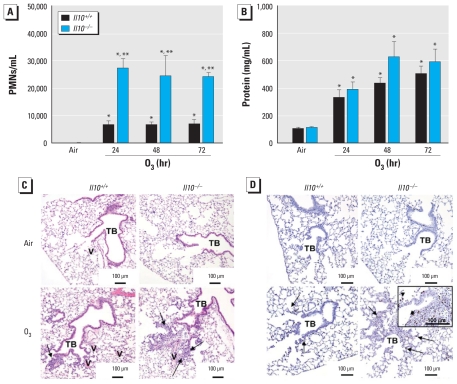
Changes in number of PMNs (*A*) and total protein concentration (*B*) recovered in BALF from *Il10*^+/+^ and *Il10*^−/−^ mice in response to air or 0.3 ppm O_3_ (means ± SE; *n* = 8/group). *p < 0.05, air versus 0.3 ppm O_3_; **p < 0.05, *Il10*^+/+^ versus *Il10*^−/−^. (*C*) H&E staining of 5-μm lung sections from *Il10*^+/+^ and *Il10*^−/−^ mice after air or 0.3 ppm O_3_ (72 hr). Arrows illustrate areas of greatest neutrophilic inflammation, particularly around terminal bronchioles (TB) and blood vessels (V). Sections are representative of mice from each treatment group. (*D*) Ki67 immunostaining of 5-μm lung sections from *Il10*^+/+^ and *Il10*^−/−^ mice after air or 0.3 ppm O_3_ (72 hr). Arrows indicate foci of cellular proliferation (arrows indicate the arrowheads shown in the inset). Sections are representative of mice from each treatment group.

O_3_-induced increases in total numbers of BALF cells were significantly greater in *Il10*^−/−^ compared with *Il10*^+/+^ mice [see Supplemental Material, Table 1 (doi:10.1289/ehp.1002182)] and largely attributable to differences in total numbers of PMNs. Compared with *Il10*^+/+^ mice, the numbers of PMNs were significantly greater (~3-fold) in *Il10*^−/−^ mice at each O_3_ exposure time point (Figure 1A). Total BALF protein concentrations also increased significantly in both genotypes after O_3_, but we found no significant differences between the two genotypes (*p* = 0.055; Figure 1B).

### Histopathological analysis of lung injury

Consistent with BALF phenotypes, we found greater O_3_-induced inflammation in perivascular, peribronchiolar, and terminal bronchial regions in hemotoxylin and eosin (H&E)-stained lung tissue sections from *Il10*^−/−^ mice compared with *Il10*^+/+^ mice (Figure 1C). Further, the density of Ki67-positive cells at distal perivascular-peribronchiolar areas and centriacinar regions was more prominent in *Il10*^−/−^ than in *Il10*^+/+^ mice exposed to O_3_, indicating enhanced cellular proliferation (Figure 1D).

### Inflammatory mediator production

**Figure 2:**
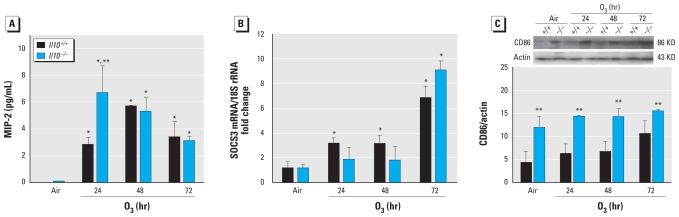
(*A*) MIP-2 protein levels in BALF recovered from *Il10*^+/+^ and *Il10*^−/−^ mice exposed to air and 0.3 ppm O_3_ (means ± SE; *n* = 4 per group). *p < 0.05, air versus O_3_; **p < 0.05, *Il10*^+/+^ versus *Il10*^−/−^. (*B*) *Socs3* mRNA expression in *Il10*^+/+^ and *Il10*^−/−^ mice in response to air or 0.3 ppm O_3_. We determined the ratio of *Socs3* to 18S RNA from whole-lung homogenates from PCR (means ± SE; *n* = 3 per group). *p < 0.05, air versus 0.3 ppm O_3_. (*C*) Lung CD86 protein expression in *Il10*^+/+^ and *Il10*^−/−^ mice exposed to air and 0.3 ppm O_3_ (means ± SE; *n* = 3–5 per group). We detected CD86 expression from whole-lung homogenates by Western blot analysis and reprobed blots with actin for normalization. +*p* < 0.05.

We did not detect *Il10* mRNA expression in *Il10*^−/−^ mouse lung homogenates after air or O_3_ exposure (data not shown). However, *Il10* mRNA was significantly elevated relative to air-exposed controls after 24-hr O_3_ (4.4-fold; *p* < 0.05) but was not significantly different between air- and O_3_-exposed *Il10*^+/+^ mice after 48- and 72-hr exposures (data not shown). Relative to respective air controls, O_3_ significantly increased mean BALF concentration of the PMN chemoattractant MIP-2 in both genotypes and was significantly higher in *Il10*^−/−^ compared with *Il10*^+/+^ mice at 24 hr (Figure 2A). However, these genotype-dependent differences were not evident at 48- and 72-hr O_3_. TNF-α and iNOS mRNA expression levels were significantly increased in both strains during O_3_, but we found no significant genotypic differences (data not shown).

### SOCS3 expression

Because SOCS3 has been proposed as a mechanism of IL-10–mediated protection against inflammatory processes in other models of lung disease (Gao and Ward 2007), we asked whether SOCS3 was differentially expressed in *Il10*^−/−^ and *Il10*^+/+^ mice. Relative to air controls, *Socs3* mRNA expression increased significantly in *Il10*^+/+^ mice after 24-, 48-, and 72-hr O_3_ (Figure 2B); *Socs3* expression in *Il10*^−/−^ mice was increased significantly only after 72-hr O_3_.

### CD86 production

Compared with *Il10*^+/+^ mice, we found significantly higher levels of lung CD86 proteins in *Il10*^−/−^ mice after air and O_3_ (Figure 2C). However, O_3_ did not increase CD86 in either genotype, and we found no interaction between genotype and exposure.

### Nuclear NF-κB activity and STAT3 activation

**Figure 3:**
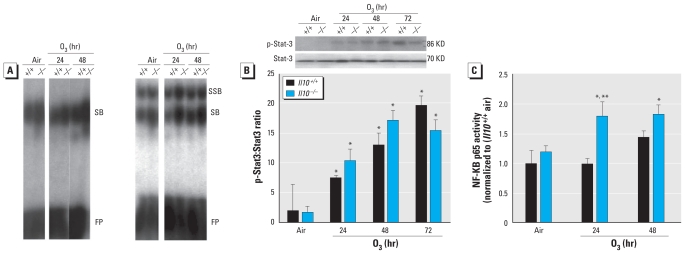
(*A*) EMSA to determine DNA binding activity of total NF-κB (top left) and specific p50 κB (top right) after 0.3 ppm O_3_ in *Il10*^+/+^ and *Il10*^−/−^ mice. Each lane represents nuclear protein pooled from three representative animals of each treatment group and was repeated three times. SB, shifted band; SSB, supershifted band; FP, free probe. Quantified p65 κB determined by ELISA is presented below (means ± SE; *n* = 3 per group). *p < 0.05, air versus 0.3 ppm O_3_; **p < 0.05, *Il10*^+/+^ versus *Il10*^−/−^. (*B*) Phosphorylated STAT3 (p-STAT3) in *Il10*^+/+^ and *Il10*^−/−^ mice in response to air or 0.3 ppm O_3_. We determined the ratio of p-STAT3 to total STAT3 from whole-lung homogenates by Western blot analysis (means ± SE; *n* = 3 per group). *p < 0.05, air versus 0.3 ppm O_3_.

To determine whether the protective effect of IL-10 may be mediated in part by differential NF-κB activity, we evaluated nuclear DNA binding activity of total NF-κB and specific p50, a subunit of NF-κB and p65 κB activity in *Il10*^+/+^ and *Il10*^−/−^ mice. Interestingly, baseline binding activity of total NF-κB and specific p50 κB was slightly greater in *Il10*^−/−^ mice than in *Il10*^+/+^ mice (Figure 3A). O_3_ effects on activation of total NF-κB and specific p50 κB binding activity were more marked in *Il10*^−/−^ mice than in *Il10*^+/+^ mice (Figure 3A). Specific p65 subunit NF-κB activity was significantly increased only in *Il10*^−/−^ mice after O_3_ (Figure 3A). The ratio of phosphorylated STAT3 (p-STAT3) to STAT3 protein in the lung increased significantly after O_3_ compared with air controls in both genotypes, signifying an increase in activated STAT3 protein in response to O_3_ (Figure 3B). However, we found no differences in O_3_-induced increases in the p-STAT3:STAT3 ratio between *Il10*^−/−^ and *Il10*^+/+^ mice.

### O_3_-induced gene expression profiles are altered in *Il10*^+/+^ compared with *Il10*^−/−^ mice

**Figure 4:**
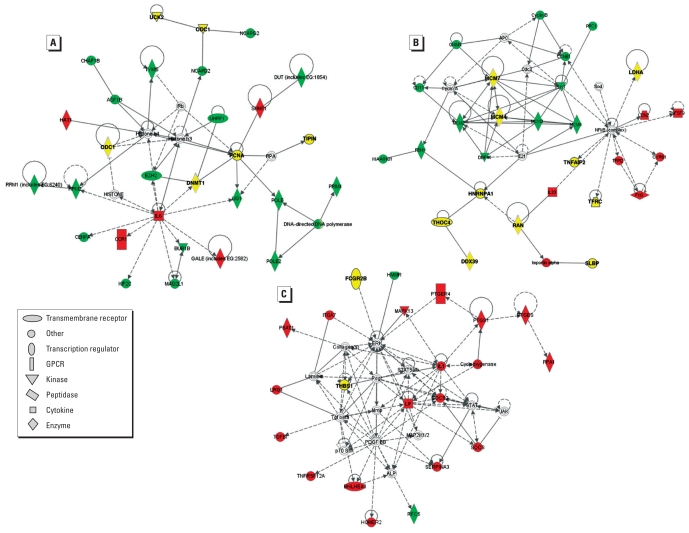
IPA illustrates the three most highly associated networks (*A*–*C*) of IL-10–dependent genes in response to O_3_. Red symbols, genes whose profiles were derived from Supplemental Material Figure 5A [see Supplemental Material, Figure 5, Table 2 (doi:10.1289/ehp.1002182)]; yellow symbols, genes with profiles that followed the pattern illustrated in Supplemental Material Figure 5B (see Supplemental Material, Figure 5, Table 2); green symbols, genes with profiles that differ between *Il10*^+/+^ and *Il10*^−/−^ mice [see Supplemental Material, Figure 5D, Table 2 (doi:10.1289/ehp.1002182)]. Gene abbreviations are identified in Supplemental Material, Table 2. GPCR, G-protein–coupled receptor.

We first used unsupervised analyses of gene expression profiles in *Il10*^−/−^ and *Il10*^+/+^ mice to provide additional insight into the means through which IL-10 protects the lung against O_3_-induced inflammation. Using k-means clustering analysis, we identified differential gene expression profile patterns between *Il10*^+/+^ and *Il10*^−/−^ mice that suggested genotype-independent and genotype-specific O_3_ effects [for gene expression clusters and gene lists, see Supplemental Material (doi:10.1289/ehp.1002182)]. IPA revealed distinct gene expression pathways for specific O_3_-induced changes that displayed similar kinetics between *Il10*^+/+^ and *Il10*^−/−^ mice [Figure 4, red shapes; see also Supplemental Material, Table 2, Figure 5A (doi:10.1289/ehp.1002182)]. For example, *Gale* (galatose-4-epimerase, UDP), *Hat1* (histone acetyl transferase 1), and *Ccr1* [chemokine (C-C motif) receptor-1] expression was altered after O_3_ (Figure 4A, red shapes). In addition, we observed O_3_-induced gene expression profile differences in *Il33* in *Il10*^−/−^ and *Il10*^+/+^ mice (Figure 4B, red shapes), *Socs3*, and *Il1* (Figure 4C, red shapes). This is consistent with Figure 2B that shows parallel increases in *Socs3* transcript level in both strains, although statistical testing revealed a significant increase in *Il10*^+/+^ mice earlier than in the *Il10*^−/−^ mice. These genes are associated with stress and inflammatory pathways [see Supplemental Material, Table 3 (doi:10.1289/ehp.1002182)]. Moreover, we found O_3_-induced gene expression patterns that were altered temporally in *Il10*^−/−^ compared with *Il10*^+/+^ mice [Figure 4, green shapes; see also Supplemental Material, Figure 5D, Table 2 (doi:10.1289/ehp.1002182)].

We also applied supervised analysis using GeneSpring to identify genes that varied statistically significantly between *Il10*^+/+^ and *Il10*^−/−^ mice during 24- to 72-hr O_3_ [see Supplemental Material, Figure 6, Table 4 (doi:10.1289/ehp.1002182)]. Genes > 2-fold higher in *Il10*^−/−^ mice than in *Il10*^+/+^ mice included those encoding CTSE, WDFY1 (WD repeat and FYVE domain containing 1), IL-1β, SAA3, nicotinamide nucleotide transhydrogenase (NNT), and peptidylglycine α-amidating monooxygenase (PAM). Many of these genes were also elucidated by unsupervised analysis [see Supplemental Material, Table 4 (doi:10.1289/ehp.1002182)]. We validated a subset of the genes (*n* = 4) listed in the Supplemental Material, Table 4 (doi:10.1289/ehp.1002182), by measuring mRNA expression in lung homogenates from air- and O_3_-exposed *Il10*^−/−^ and *Il10*^+/+^ mice. We found that mRNA expression of *Ctse* (24 hr), *Wdfy1* (24, 48 hr), *Saa3* (48 hr), and *S100a14* (S100 calcium binding protein A14; 24, 48, and 72 hr) was significantly (*p* < 0.05) greater in *Il10*^−/−^ than in *Il10*^+/+^ lung homogenates [see Supplemental Material, Figure 7 (doi:10.1289/ehp.1002182)].

## Discussion

We investigated the mechanisms of IL-10–mediated reductions in O_3_-induced airway neutrophilia in mice. Targeted deletion of *Il10* significantly enhanced O_3_-induced pulmonary cellular inflammation, as indicated by significant differences in numbers of infiltrating neutrophils and enhanced cellular proliferation in centriacinar regions compared with *Il10*^+/+^ controls. Nuclear activity of NF-κB and expression of CD86 proteins were also greater in lungs of *Il10*^−/−^ mice. Furthermore, MIP-2 protein was significantly elevated in response to 24-hr O_3_ and was exacerbated in *Il10*^−/−^ compared with *Il10*^+/+^ mice. Results support a role for IL-10 in protection against O_3_-induced inflammation associated with diminished NF-κB activity and MIP-2 production. Nonbiased (visual, unsupervised) and supervised (ANOVA) gene array analysis identified novel cellular targets that may contribute to the protective mechanism of IL-10 in O_3_-induced inflammation.

O_3_-induced increases in BALF neutrophils and lung cell proliferation were significantly enhanced in *Il10*^−/−^ mice relative to *Il10*^+/+^ wild-type controls, but the lung permeability response to O_3_ was not significantly different between the two genotypes. Interestingly, Reinhart et al. (1999) found that intratracheal instillation of recombinant IL-10 into Sprague-Dawley rats significantly attenuated by 25% protein permeability responses induced by acute O_3_ exposure (0.8 ppm, 3 hr) compared with controls. An explanation for the disparate observations is not entirely clear, but the role of IL-10 in the hyperpermeability response may depend on the concentration and duration of exposure to O_3_ (0.8 ppm for 3 hr compared with 0.3 ppm for 24, 48, or 72 hr) or may be species specific. Moreover, these results are consistent with previous studies suggesting that inflammatory and permeability responses in this model are regulated through different mechanisms (e.g., Cho et al. 2007).

Upregulation of the PMN chemoattractant MIP-2 has been suggested to regulate initial neutrophilic infiltration in response to O_3_. O_3_-induced increases in MIP-2 were potentiated in the absence of *Il10* at 24 hr before the peak PMN influx and therefore may be a mechanism through which IL-10 protects against O_3_-induced inflammation and cell proliferation. Similarly, neutralization of IL-10 prior to LPS administration can attenuate production of MIP-2 and associated neutrophilia (Standiford et al. 1995). We observed no significantly different O_3_-induced changes in TNF-α and iNOS between *Il10*^−/−^ and *Il10*^+/+^ mice, despite genotype-specific differences in NF-κB after O_3_. These results suggest that O_3_-induced increases in TNF-α and iNOS, which are thought to be NF-κB dependent (e.g., Cho et al. 2007), are not modulated by IL-10. These results are inconsistent with those of Lang et al. (2002) and Standiford et al. (1995), who found that TNF-α production is IL-10 dependent in responses to endotoxin. Collectively, these studies may suggest important differences in pulmonary cellular responses to endotoxin and O_3_ and warrant further investigation.

We also found that lung CD86 protein levels were enhanced in *Il10*^−/−^ compared with *Il10*^+/+^ mice, independent of O_3_ exposure. Soltys et al. (2002) showed that CD86 in alveolar macrophages was significantly enhanced in *Il10*^−/−^ mice at baseline and suggested that low levels of alveolar macrophage costimulatory molecule expression are maintained at least in part by endogenous IL-10 activity. Koike et al. (2001) found that approximately 8% of alveolar macrophages obtained from rats exposed to 1 ppm O_3_ for 3 days were CD86 positive and suggested that small increases in costimulatory activity may be sufficient to regulate O_3_-induced immune responses. Our data similarly indicate that *Il10* deficiency permits endogenous CD86 expression, which could explain why we did not detect subtle increases in CD86 in *Il10*^−/−^ mice after O_3_ exposure.

To investigate the potential signaling pathway through which IL-10 protects against O_3_-induced inflammation, we evaluated NF-κB activation in *Il10*^+/+^ and *Il10*^−/−^ mice. NF-κB is a nuclear protein that regulates transcription of many gene products that activate major pulmonary inflammatory pathways, including responses to O_3_ (Cho et al. 2007). IL-10 inhibits NF-κB (Saadane et al. 2005; Spight et al. 2005). We found that O_3_-induced p50 levels and p65 κB binding activity were significantly greater in *Il10*^−/−^ than in *Il10*^+/+^ mice. The mechanism through which IL-10 inhibits NF-κB may occur indirectly, such as through inhibition of inhibitory kappa kinase, as demonstrated in response to LPS (Saadane et al. 2005; Spight et al. 2005).

STAT3 was associated with IL-10–mediated inhibition of alveolar macrophage activation by LPS (Berlato et al. 2002). *Stat3* has also been shown to mediate O_3_-induced inflammation (Laskin et al. 2002) and pulmonary inflammation and hyperresponsiveness associated with asthmatic responses (Corry 2002). Interestingly, we found no significant genotype-specific differences in the p-STAT3:STAT3 ratio (Figure 3B). Similarly, gene array analysis did not detect an interaction among O_3_ exposure, *Il10* deficiency, and *Stat3* gene expression at these time points. These results suggest that O_3_-induced STAT3 activation occurs in an IL-10–independent manner.

To further elucidate the potential mechanism through which IL-10 protects the lung against O_3_-induced inflammation, we used k-means clustering and Ingenuity analyses of microarray expression data. We identified three distinct clusters of molecules, representing three pathways and numerous gene targets. Several genes identified in this analysis (e.g., *Il6*, *Il1ra*, *Il1*) contribute to O_3_-induced pulmonary inflammation in mice (Figure 4).

Other identified genes represent novel targets for O_3_-induced inflammation. For example, both gene expression analyses identified *Ctse* as differentially expressed between genotypes after exposure to O_3_. *Ctse* is a an intracellular aspartic protease that is found in immune system cells such as dendritic cells and macrophages and has been implicated in major histocompatibility complex (MHC) class II pathway antigen processing (e.g., Zaidi and Kalbacher 2008). Although *Ctse* has not been associated with O_3_-induced inflammation, MHC class II molecules have been implicated recently in the PMN response to O_3_ exposure in the mouse (Bauer et al. 2009), and it is plausible that CTSE could also be involved in this pathway. SAA and S100A14 have been implicated in inflammatory processes (e.g., Han et al. 2007) or inflammation-related diseases (Chen et al. 2009) and thus provide a rationale for a role in O_3_-induced inflammation.

Our gene array analysis identified *Socs3* as being induced in response to O_3_, compared with air controls. RT-PCR analyses confirmed O_3_-induced upregulation of *Socs3* and identified a small but statistically significant difference in expression kinetics between *Il10*^+/+^ and *Il10*^−/−^ mice. Members of the SOCS signaling family, including SOCS3, regulate activation and function of STAT3 (Gao and Ward 2007). Emerging data on activation and function of STAT3 and SOCS3 in the lung during acute inflammation suggest that these molecules may regulate pulmonary inflammation (Gao and Ward 2007), and they have specifically been implicated in allergic airway inflammation (Paul et al. 2009). Given that SOCS3 is a proximal mediator associated with IL-10 ligand binding, the role of SOCS3 in O_3_-induced inflammation necessitates further investigation to determine its specific contribution to the adaptive/innate immune response.

The array analysis implicated a number of novel genes for response to O_3_ in *Il10*^+/+^ and *Il10*^−/−^ mice, including *Il33* and *Hat1*. IL-33 is a member of the IL-1 superfamily and is expressed on epithelial cells. This cytokine has been identified in joints of arthritis patients (Palmer et al. 2009) and is implicated in development and regulation of type 2 T-helper cell–dependent immune responses in sites of mucosal immunity, which may suggest a link between innate and adaptive immune responses after O_3_ exposure (Saenz et al. 2008). HAT1, an acetylation enzyme, controls access to DNA transcription by controlling histone protein activation during chromatin assembly (Parthun 2007). Although HAT1 expression has been linked to inflammatory mediators such as interferon-γ and TNF-α (Keslacy et al. 2007), the specific role of HAT1 in response to O_3_ remains unclear.

## Conclusion

This study supports the hypothesis that IL-10 protects against O_3_-induced lung inflammation and identified several potential mechanisms involved in this response. IL-10 deficiency enhanced O_3_-induced neutrophilic inflammation and injury at the centriacinar region of the lung. Results also suggest that in response to O_3_ the effect of IL-10 may be mediated, in part, via modulation of NF-κB, MIP-2, and CD86. Gene array analysis identified three IL-10–mediated expression pathways with genes known previously to be affected by O_3_ and novel genes that may contribute significantly to the pathogenesis of O_3_-induced pulmonary inflammation. The present investigation identified novel molecular mechanisms of pulmonary O_3_ toxicity, which may provide possible therapeutic targets for attenuating the effects of O_3_ in susceptible individuals.

## References

[b1-ehp-118-1721] Akdis CA, Joss A, Akdis M, Blaser K (2001). Mechanism of IL-10-induced T cell inactivation in allergic inflammation and normal response to allergens. Int Arch Allergy Immunol.

[b2-ehp-118-1721] American Physiological Society (2002). Appendix C. Guiding Principles for Research Involving Animals and Human Beings. Recommendations From The Revised Declaration of Helsinki by the World Medical Association Regarding Human Subjects.

[b3-ehp-118-1721] Bauer AK, Travis EL, Malhotra SS, Rondini EA, Walker C, Cho HY (2009). Identification of novel susceptibility genes in ozone-induced inflammation in mice. Eur Respir J.

[b4-ehp-118-1721] Berlato C, Cassatella MA, Kinjyo I, Gatto L, Yoshimura A, Bazzoni F (2002). Involvement of suppressor of cytokine signaling-3 as a mediator of the inhibitory effects of IL-10 on lipopolysaccharide-induced macrophage activation. J Immunol.

[b5-ehp-118-1721] Bhalla DK, Reinhart PG, Bai C, Gupta SK (2002). Amelioration of ozone-induced lung injury by antitumor necrosis factor-alpha. Toxicol Sci.

[b6-ehp-118-1721] Chatterjee R, Batra J, Kumar A, Mabalirajan U, Nahid S, Niphadkar PV (2005). Interleukin-10 promoter polymorphisms and atopic asthma in North Indians. Clin Exp Allergy.

[b7-ehp-118-1721] Chen H, Yu D, Luo A, Tan W, Zhang C, Zhao (2009). Functional role of S100A14 genetic variants and their association with esophageal squamous cell carcinoma. Cancer Res.

[b8-ehp-118-1721] Cho HY, Morgan DL, Bauer AK, Kleeberger SR (2007). Signal transduction pathways of tumor necrosis factor--mediated lung injury induced by ozone in mice. Am J Respir Crit Care Med.

[b9-ehp-118-1721] Cho HY, Zhang LY, Kleeberger SR (2001). Ozone-induced lung inflammation and hyperreactivity are mediated via tumor necrosis factor-alpha receptors. Am J Physiol Lung Cell Mol Physiol.

[b10-ehp-118-1721] Corry DB (2002). Emerging immune targets for the therapy of allergic asthma. Nat Rev Drug Discov.

[b11-ehp-118-1721] Cunha FQ, Moncada S, Liew FY (1992). Interleukin-10 (IL-10) inhibits the induction of nitric oxide synthase by interferon-gamma in murine macrophages. Biochem Biophys Res Commun.

[b12-ehp-118-1721] Depuydt PO, Lambrecht BN, Joos GF, Pauwels RA (2002). Effect of ozone exposure on allergic sensitization and airway inflammation induced by dendritic cells. Clin Exp Allergy.

[b13-ehp-118-1721] Ding L, Linsley PS, Huang LY, Germain RN, Shevach EM (1993). IL-10 inhibits macrophage costimulatory activity by selectively inhibiting the up-regulation of B7 expression. J Immunol.

[b14-ehp-118-1721] Donnelly RP, Dickensheets H, Finbloom DS (1999). The interleukin-10 signal transduction pathway and regulation of gene expression in mononuclear phagocytes. J Interferon Cytokine Res.

[b15-ehp-118-1721] Driscoll KE, Simpson L, Carter J, Hassenbein D, Leikauf GD (1993). Ozone inhalation stimulates expression of a neutrophil chemotactic protein, macrophage inflammatory protein 2. Toxicol Appl Pharmacol.

[b16-ehp-118-1721] Fakhrzadeh L, Laskin JD, Laskin DL (2002). Deficiency in inducible nitric oxide synthase protects mice from ozone-induced lung inflammation and tissue injury. Am J Respir Cell Mol Biol.

[b17-ehp-118-1721] Flores Villanueva PO, Reiser H, Stadecker MJ (1994). Regulation of T helper cell responses in experimental murine schistosomiasis by IL-10. Effect on expression of B7 and B7–2 costimulatory molecules by macrophages. J Immunol.

[b18-ehp-118-1721] Gao H, Ward PA (2007). STAT3 and suppressor of cytokine signaling 3: potential targets in lung inflammatory responses. Expert Opin Ther Targets.

[b19-ehp-118-1721] Han CY, Subramanian S, Chan CK, Omer M, Chiba T, Wight TN (2007). Adipocyte-derived serum amyloid A3 and hyaluronan play a role in monocyte recruitment and adhesion. Diabetes.

[b20-ehp-118-1721] Higgins SC, Lavelle EC, McCann C, Keogh B, McNeela E, Byrne P (2003). Toll-like receptor 4-mediated innate IL-10 activates antigen-specific regulatory T cells and confers resistance to Bordetella pertussis by inhibiting inflammatory pathology. J Immunol.

[b21-ehp-118-1721] Huaux F, Louahed J, Hudspith B, Meredith C, Delos M, Renauld JC (1998). Role of interleukin-10 in the lung response to silica in mice. Am J Respir Cell Mol Biol.

[b22-ehp-118-1721] Inoue H, Aizawa H, Nakano H, Matsumoto K, Kuwano K, Nadel JA (2000). Nitric oxide synthase inhibitors attenuate ozone-induced airway inflammation in guinea pigs. Possible role of interleukin-8. Am J Respir Crit Care Med.

[b23-ehp-118-1721] Institute of Laboratory Animal Resources (1996). Guide for the Care and Use of Laboratory Animals.

[b24-ehp-118-1721] Jakab GJ, Spannhake EW, Canning BJ, Kleeberger SR, Gilmour MI (1995). The effects of ozone on immune function. Environ Health Perspect.

[b25-ehp-118-1721] Jerrett M, Burnett RT, Pope CA, Ito K, Thurston G, Krewski D (2009). Long-term exposure to ozone and mortality. N Engl J Med.

[b26-ehp-118-1721] Johnston RA, Schwartzman IN, Flynt L, Shore SA (2005). Role of interleukin-6 in murine airway responses to ozone. Am J Physiol Lung Cell Mol Physiol.

[b27-ehp-118-1721] Keslacy S, Tliba O, Baidouri H, Amrani Y (2007). Inhibition of tumor necrosis factor-alpha-inducible inflammatory genes by interferon-gamma is associated with altered nuclear factor-kappaB transactivation and enhanced histone deacetylase activity. Mol Pharmacol.

[b28-ehp-118-1721] Kleeberger SR, Reddy SP, Zhang LY, Cho HY, Jedlicka AE (2001). Toll-like receptor 4 mediates ozone-induced murine lung hyperpermeability via inducible nitric oxide synthase. Am J Physiol Lung Cell Mol Physiol.

[b29-ehp-118-1721] Koike E, Kobayashi T, Shimojo N (2001). Ozone exposure enhances expression of cell-surface molecules associated with antigen-presenting activity on bronchoalveolar lavage cells in rats. Toxicol Sci.

[b30-ehp-118-1721] Lang R, Patel D, Morris JJ, Rutschman RL, Murray PJ (2002). Shaping gene expression in activated and resting primary macrophages by IL-10. J Immunol.

[b31-ehp-118-1721] Laskin DL, Fakhrzadeh L, Heck DE, Gerecke D, Laskin JD (2002). Upregulation of phosphoinositide 3-kinase and protein kinase B in alveolar macrophages following ozone inhalation. Role of NF-kappaB and STAT-1 in ozone-induced nitric oxide production and toxicity. Mol Cell Biochem.

[b32-ehp-118-1721] McConnell R, Berhane K, Gilliland F, London SJ, Islam T, Gauderman WJ (2002). Asthma in exercising children exposed to ozone: a cohort study. Lancet.

[b33-ehp-118-1721] Mudway IS, Kelly FJ (2000). Ozone and the lung: a sensitive issue. Mol Aspects Med.

[b34-ehp-118-1721] Neuhaus-Steinmetz U, Uffhausen F, Herz U, Renz H (2000). Priming of allergic immune responses by repeated ozone exposure in mice. Am J Respir Cell Mol Biol.

[b35-ehp-118-1721] Palmer G, Talabot-Ayer D, Lamacchia C, Toy D, Seemayer CA, Viatte S (2009). Inhibition of interleukin-33 signaling attenuates the severity of experimental arthritis. Arthritis Rheum.

[b36-ehp-118-1721] Parthun MR (2007). Hat1: the emerging cellular roles of a type B histone acetyltransferase. Oncogene.

[b37-ehp-118-1721] Paul B, Mishra V, Chaudhury B, Awasthi A, Das AB, Saxena U (2009). Status of Stat3 in an ovalbumin-induced mouse model of asthma: analysis of the role of Socs3 and IL-6. Int Arch Allergy Immunol.

[b38-ehp-118-1721] Plopper CG, Hatch GE, Wong V, Duan X, Weir AJ, Tarkington BK (1998). Relationship of inhaled ozone concentration to acute tracheobronchial epithelial injury, site-specific ozone dose, and glutathione depletion in rhesus monkeys. Am J Respir Cell Mol Biol.

[b39-ehp-118-1721] Reinhart PG, Gupta SK, Bhalla DK (1999). Attenuation of ozone-induced lung injury by interleukin-10. Toxicol Lett.

[b40-ehp-118-1721] Saadane A, Soltys J, Berger M (2005). Role of IL-10 deficiency in excessive nuclear factor-kappaB activation and lung inflammation in cystic fibrosis transmembrane conductance regulator knockout mice. J Allergy Clin Immunol.

[b41-ehp-118-1721] Saenz SA, Taylor BC, Artis D (2008). Welcome to the neighborhood: epithelial cell-derived cytokines license innate and adaptive immune responses at mucosal sites. Immunol Rev.

[b42-ehp-118-1721] Schippers EF, van ‘t Veer C, van Voorden S, Martina CA, Huizinga TW, le Cessie S (2005). IL-10 and toll-like receptor-4 polymorphisms and the in vivo and ex vivo response to endotoxin. Cytokine.

[b43-ehp-118-1721] Soltys J, Bonfield T, Chmiel J, Berger M (2002). Functional IL-10 deficiency in the lung of cystic fibrosis (cftr(−/−)) and IL-10 knockout mice causes increased expression and function of B7 costimulatory molecules on alveolar macrophages. J Immunol.

[b44-ehp-118-1721] Spight D, Zhao B, Haas M, Wert S, Denenberg A, Shanley TP (2005). Immunoregulatory effects of regulated, lung-targeted expression of IL-10 in vivo. Am J Physiol Lung Cell Mol Physiol.

[b45-ehp-118-1721] Standiford TJ, Strieter RM, Lukacs NW, Kunkel SL (1995). Neutralization of IL-10 increases lethality in endotoxemia. Cooperative effects of macrophage inflammatory protein-2 and tumor necrosis factor. J Immunol.

[b46-ehp-118-1721] Tarzi M, Klunker S, Texier C, Verhoef A, Stapel SO, Akdis CA (2006). Induction of interleukin-10 and suppressor of cytokine signalling-3 gene expression following peptide immunotherapy. Clin Exp Allergy.

[b47-ehp-118-1721] Toward TJ, Broadley KJ (2002). Airway function, oedema, cell infiltration and nitric oxide generation in conscious ozone-exposed guinea-pigs: effects of dexamethasone and rolipram. Br J Pharmacol.

[b48-ehp-118-1721] Zaidi N, Kalbacher H (2008). Cathepsin E: a mini review. Biochem Biophys Res Commun.

